# PEGylated Serp-1 Markedly Reduces Pristane-Induced Experimental Diffuse Alveolar Hemorrhage, Altering uPAR Distribution, and Macrophage Invasion

**DOI:** 10.3389/fcvm.2021.633212

**Published:** 2021-02-16

**Authors:** Qiuyun Guo, Jordan R. Yaron, John W. Wallen, Kyle F. Browder, Ryan Boyd, Tien L. Olson, Michelle Burgin, Peaches Ulrich, Emily Aliskevich, Lauren N. Schutz, Petra Fromme, Liqiang Zhang, Alexandra R. Lucas

**Affiliations:** ^1^Center for Personalized Diagnostics and Center for Immunotherapy, Vaccines and Virotherapy, The Biodesign Institute, Arizona State University, Tempe, AZ, United States; ^2^Department of Oncology, Tongji Hospital, Tongji Medical College, Huazhong University of Science and Technology, Wuhan, China; ^3^Exalt Therapeutics LLC, Las Vegas, NV, United States; ^4^Center for Applied Structural Discovery, The Biodesign Institute, Arizona State University, Tempe, AZ, United States

**Keywords:** lupus, diffuse alveolar hemorrhage, immune modulator, Serp-1, inflammation, recombinant protein therapeutic, vasculitis

## Abstract

Diffuse alveolar hemorrhage (DAH) is one of the most serious clinical complications of systemic lupus erythematosus (SLE). The prevalence of DAH is reported to range from 1 to 5%, but while DAH is considered a rare complication there is a reported 50–80% mortality. There is at present no proven effective treatment for DAH and the therapeutics that have been tested have significant side effects. There is a clear necessity to discover new drugs to improve outcomes in DAH. *Ser*ine *p*rotease *in*hibitors, *serpins*, regulate thrombotic and thrombolytic protease cascades. We are investigating a Myxomavirus derived immune modulating serpin, Serp-1, as a new class of immune modulating therapeutics for vasculopathy and lung hemorrhage. Serp-1 has proven efficacy in models of herpes virus-induced arterial inflammation (vasculitis) and lung hemorrhage and has also proved safe in a clinical trial in patients with unstable coronary syndromes and stent implant. Here, we examine Serp-1, both as a native secreted protein expressed by CHO cells and as a polyethylene glycol modified (PEGylated) variant (Serp-1m5), for potential therapy in DAH. DAH was induced by intraperitoneal (IP) injection of pristane in C57BL/6J (B6) mice. Mice were treated with 100 ng/g bodyweight of either Serp-1 as native 55 kDa secreted glycoprotein, or as Serp-1m5, or saline controls after inducing DAH. Treatments were repeated daily for 14 days (6 mice/group). Serp-1 partially and Serp-1m5 significantly reduced pristane-induced DAH when compared with saline as assessed by gross pathology and H&E staining (Serp-1, *p* = 0.2172; Serp-1m5, *p* = 0.0252). Both Serp-1m5 and Serp-1 treatment reduced perivascular inflammation and reduced M1 macrophage (Serp-1, *p* = 0.0350; Serp-1m5, *p* = 0.0053), hemosiderin-laden macrophage (Serp-1, *p* = 0.0370; Serp-1m5, *p* = 0.0424) invasion, and complement C5b/9 staining. Extracellular urokinase-type plasminogen activator receptor positive (uPAR+) clusters were significantly reduced (Serp-1, *p* = 0.0172; Serp-1m5, *p* = 0.0025). Serp-1m5 also increased intact uPAR+ alveoli in the lung (*p* = 0.0091). In conclusion, Serp-1m5 significantly reduces lung damage and hemorrhage in a pristane model of SLE DAH, providing a new potential therapeutic approach.

## Introduction

Systemic lupus erythematosus (SLE), or lupus, is an autoimmune disease characterized by immune cell hyperactivity, production of antibodies against self-antigens, such as double-stranded (ds) DNA, histones, and ribonucleoprotein (RNP). The etiology of SLE is only partially defined and has been linked to abnormal genetic, hormonal, and environmental responses (1–4). The incidence of this disease is 20–70 per 100,000 people, and the incidence in women is 6–10 times that of men. Patients with SLE have a wide range of clinical symptoms, including skin rash, nephritis, non-erosive arthritis, serositis, cardiovascular involvement, hematological, and respiratory disorders (with pulmonary fibrosis and hypertension). In some cases (1) The most serious clinical manifestation of SLE is diffuse alveolar hemorrhage (DAH), with prevalence ranging from 1 to 5%, but causing >50–80% mortality in affected SLE patients (2–4). Lupus DAH is characterized by neutrophilic capillaritis with destruction of alveolar septae and infiltration of hemosiderin-laden macrophages (5–8). The current treatment options for DAH include steroids, cyclophosphamide, rituximab, methotrexate, azathioprine, respiratory support, and *among others*. The efficacy of these treatments is limited and there are many significant side effects, that include hypertension, diabetes, osteoporosis, adrenal suppression, infertility, pulmonary fibrosis, hepatotoxicity, and risk for future malignancies. Therefore, there is an urgent, unmet need for new drugs to improve treatment for DAH.

Pristane (2, 6, 10, and 14 tetramethylpentadecane, TMPD) is an isoprenoid alkane found at high concentration in mineral oil, and in low concentration in vegetables (9). It is also found in the liver of some sharks (10). Intraperitoneal (IP) injections of pristane can induce in mice a wide range of autoantibodies specific to, or associated with, SLE (11, 12), making pristane an accepted method to establish mouse models of SLE. IP injection of pristane in C57BL/6J (B6) mice causes severe alveolar hemorrhage within 2 weeks, manifested by alveolar and perivascular inflammation (capillaritis, small vessel vasculitis), endothelial injury and hemorrhage (7, 13–15). Many previous studies have proven that this model can closely simulate the pathological process of DAH.

Serp-1 is a purified 55 kDa secreted glycoprotein originally derived from MYXV, belonging to the SERPIN superfamily. Our previous research has demonstrated that purified Serp-1 protein treatment is beneficial in a wide range of immune mediated disorders, from arthritis to vasculitis to transplant (16–21). Serp-1 reduces macrophage cell infiltration into transplanted hearts, kidneys and aorta in rodent models, with improved histopathological evidence of acute and chronic rejection (16, 17, 22). In a mouse model of inflammatory vasculitis induced by mouse gamma herpesvirus-68 (MHV-68) infection in interferon gamma receptor deficient mice (IFNγR^−/−^) and also in an aortic transplant model, Serp-1 significantly reduced arterial inflammation and plaque growth. Additionally, Serp-1 treatment reduced lung hemorrhage and consolidation and improved survival in mouse gamma herpesvirus-68 (MHV68) infected mice, a model for inflammatory vasculitis and lethal lung hemorrhage (20, 21). In clinical trials, Serp-1 treatment proved safe and significantly reduced markers for myocardial damage after coronary stent implant in phase I and IIa clinical trials in patients with unstable angina pectoris or non-ST elevation myocardial infarction (NSTEMI), with no significant major adverse reactions (MACE = 0) and no neutralizing antibody detected (23).

Urokinase type plasminogen activator (uPA) binds to the uPA receptor (uPAR). The uPA/uPAR complex sits at the leading, or invading, edge of inflammatory macrophage cells. In addition to a role in thrombolysis, the uPA/uPAR complex also activates plasmin which in turn activates matrix metalloproteinases (MMPs). MMPs break down connective tissue (collagen and elastin), to allow immune cells to infiltrate tissues. The uPA/uPAR complex thus functions both in fibrinolysis and in inflammatory cell activation and invasion, the latter being considered the predominant function. Serp-1 binds and inhibits thrombolytic protease, tissue- and urokinase-type plasminogen activators (tPA and uPA, respectively) as well as thrombotic proteases, thrombin and factor Xa. Serp-1 binds to the uPA/ uPAR complex on the macrophage plasma membrane surface (24, 25). Serp-1 inhibition of macrophage migration is dependent upon uPAR expression *in vitro* in monocytes and *in vivo* in the aortic transplant model. Serp-1 efficacy was previously found to be dependent on uPAR expression in the donor aorta in aortic transplant models in mice (18, 24, 26). Serp-1 treatment is thus projected to either reduce excess thrombolysis, or to rebalance an imbalance in both thrombotic and thrombolytic cascades, and to reduce inflammation in this SLE lung hemorrhage model. In previous studies another member of the Serpin superfamily, Alpha-1-antitrypsin (AAT), has shown anti-inflammatory and immunomodulatory functions, inhibiting the activation and recruitment of inflammatory cells when given for 1 week prior to pristane injection. Human AAT (hAAT) reduced the severity of DAH in B6 mice; hAAT transgenic mice completely prevented DAH induced by pristane (27).

The half-life for Serp-1 in circulating blood was ~20 min in clinical trial up to 1.36 days in mouse and rabbit models, and is dependent upon the model examined (23, 28). PEGylation has been demonstrated to improve the half-life and reduce antigenicity in prior work with other proteins (29). In this study, we examined treatment with either PEGylated Serp-1, here termed Serp-1m5, or with the native non-PEGylated secreted Serp-1 in the SLE DAH mouse model for efficacy and compared to Saline control alone. Based on prior studies, we have postulated that Serp-1 will prove effective and safe for the treatment of DAH.

## Materials and Methods

### Proteins and Chemicals

Serp-1 (m008.1L; NCBI Gene ID# 932146) was expressed in a Chinese hamster ovary (CHO) cell line (Viron Therapeutics Inc., London, ON, CA). The Serp-1 protein used in this research is GMP-compliant and purified by continuous chromatographic separation. The purity of Serp-1 is >95%, as determined by Coomassie stained SDS-PAGE and reverse-phase HPLC. Serp-1 was endotoxin-free by LAL (limulus amebocyte lysate) assay. Serp-1 was incubated with mPEG-NHS (5 K) (Nanocs Inc., #PG1-SC-5k-1, NY) in PBS buffer (pH 7.8) at 4°C overnight to modify the protein according to standard PEGylation protocols. PEGylated Serp-1 (Serp-1m5) was purified by FPLC using an ÄKTA pure protein purification system with Superdex-200.

Hematoxylin and eosin for H&E staining and trichrome reagents were from Sigma-Aldrich. Information about each antibody used for immunohistochemical staining in this study is provided below when first mentioned.

### Animals

All animal procedures in this study were approved by the Institutional Animal Care and Use Committee of Arizona State University under protocol #20-1761R and conform to national and international guidelines for animal care. Eighteen wild-type female C57BL6/J mice aged 6–8 weeks old were treated with pristane. Female mice are reported in prior studies to be preferred for the development of DAH model (13, 27). Each mouse was injected with 0.5 ml of pristane (Sigma-Aldrich) intraperitoneally (IP) at day 0. These mice were then randomly divided into three groups, i.e., saline, Serp-1 or Serp-1m5 treatment groups (6 mice each group, *n* = 6). Six normal mice were also examined, without pristane or Serp-1, and six mice had Serp-1 treatment without pristane. No adverse effects were seen [Toxicity for Serp-1 has been extensively tested and proven to be minimal in preclinical and clinical trials, as previously reported (16–21, 23–26)]. Each mouse was given one IP injection of 100 μL saline or 100 ng/g bodyweight of clinical grade Serp-1 or Serp-1m5 in 100 μL of Saline after pristane induction. The treatments were repeated every day until the 14th day. The mice were euthanized by CO_2_ asphyxiation on the 15th day and lung tissues were divided; one half was frozen at −80°C for later protein analysis, and one half fixed in 10% neutral-buffered formalin for at least 3 days before processing and paraffin embedding. There were no early deaths or complications in any treatment group.

### Lung Pathological Evaluation

DAH in lung specimens was initially assessed by gross observation of excised lungs, prior to either fixation or freezing. Lung tissues were fixed in 10% neutral-buffered formalin after collection and then processed in a Leica TP1050 tissue processor and embedded in paraffin with a Leica EG1160 embedding station, as previously described (21–26). Tissue blocks were cut into 5 μm sections using a Leica RM2165 microtome.

Sections were stained with hematoxylin and eosin (H&E) and by Masson's trichrome using standard procedures, as previously described (21–27, 30). DAH was classified into three degrees of severity according to the percentage of hemorrhage on H&E stained sections as assessed by a blinded histological analysis of DAH score as follows: (1) No hemorrhage (0%); (2) Partial hemorrhage (25–75%); (3) Complete hemorrhage (75–100%). Prussian blue staining (Electron Microscopy Science company) was performed with standard protocol to analyze the hemorrhage status.

Sections were additionally stained for immunohistochemical analysis (IHC) for CD3 (Abcam, ab6590, 1:100), CD4 (Abcam, ab183685, 1:1,000), Ly6G (Invitrogen, 14-5931-82, 1:100), arginase-1 (Cell Signaling, 93668, 1:200), iNOS (Abcam, ab15323, 1:100), C5b/9 antibody (Abcam, ab 55811), and uPAR (R&D Systems, AF534,1:100). HRP-conjugated secondary antibodies against rabbit or goat IgG were applied at a dilution of 1:500 for 1 h at room temperature. HRP-conjugated secondary antibody given alone without primary antibody was used as negative control for each stain. Antigens were revealed with ImmPACT DAB (Vector Labs, USA), counterstained with Gil's formula #3 Hematoxylin and mounted with Cytoseal XYL.

Slides were examined and images collected as objective-calibrated TIFFs on an Olympus BX51 upright microscope equipped with an Olympus DP74 color CMOS high-resolution camera operated by cellSens Dimensions v1.16. Images (Olympus, Waltham, MA, USA). Images were subsequently analyzed live and processed in ImageJ/FIJI. Positively stained cells were counted per high power field for each group; three high power fields examined per mouse and lung section.

### Lung Tissue Protein Extraction and Analysis

For each mouse, lungs were collected after euthanasia at 15 days post-pristane injection. One lung was fixed in neutral buffered formalin for later histological analysis. The other lung was frozen at −80°C for later biochemical assays. Half of the collected frozen tissue was homogenized as whole lung tissue with a blade homogenizer into 400 μL RIPA with EDTA buffer containing 1 × protease inhibitor cocktail (Bimake, Houston, TX, USA, #B14001) and 1 mM phenylmethanesulfonyl fluoride (PMSF) on ice. Homogenized samples were rotated at 4°C for 1 h and centrifuged at 13,000 × g for 15 min at 4°C. Supernatant containing total protein was transferred to a new tube for ELISA assays.

Half of the frozen lung tissue was homogenized into 400 μL PBS buffer containing protease inhibitors cocktail, PMSF, and 1 mM EDTA. Homogenized samples were processed by two cycles freeze-thaw, followed by centrifugation at 13,000 × g at 4°C for 15 min. Supernatant without membrane proteins was then collected for cell membrane free soluble uPAR (csuPAR) protein analysis.

### Elisa Assays

uPAR (R&D Systems, DY531) levels in lung tissues were quantified with ELISA kits following manufacturer's instructions. Quantified protein level of lung tissue was normalized to total protein, which was determined with the BCA (bicinchoninic acid) protein assay kit (Thermo Fisher Scientific, #23227).

### Mass Spectrometry Analysis of C3 Binding by Serp-1

For Serp-1 interactome analysis, 100 μL of streptavidin magnetic beads (Thermo Scientific, #88816) were washed, resuspended in 1 mL EBC buffer (50 mM Tris-HCl, 120 mM NaCl, 0.5% NP-40, pH 8.0) with 1.0% BSA. Ten microliters of biotin-labeled Serp-1 antibody (AxB7.9-biotin) was added and the mixture rotated at RT (20°C) for 2 h. Beads were washed in EBC, resuspended in 500 μL EBC buffer with 2% BSA and 20 μg Serp-1 added, and incubated with 250 μL plasma at 4°C overnight followed by buffer wash. Total binding proteins were collected by boiling the beads with 40 μL of 6 × SDS reducing dye. SDS-PAGE was performed, and bands cut from gel for interactome analysis by mass spectrometry (MS) at the ASU/Biodesign MS center.

### Flow Cytometry Analysis of Splenocytes

Spleens were isolated from mice and cells dissociated in ice-cold RPMI-1640 containing 20% FBS using a 70 μm cell strainer for immediate Flow cytometry analysis. Red blood cells were lysed using RBC lysis buffer (155 mM NH_4_Cl, 12 mM NaHCO_3_, 0.1 mM EDTA) for 10 min at room temperature and pelleted splenocytes were washed with RPMI-1640 containing 20% FBS. Splenocytes were deposited into 96-well round-bottom polystyrene plates (10^6^ splenocytes per well). Cells were either directly stained as follows or stimulated with Cell Activation Cocktail (Biolegend) in the presence of 1X Brefeldin A (Biolegend) for 90 min prior to staining. Cells were blocked on ice with TruStain FcX anti-mouse CD16/32 Fc receptor blocker (Biolegend) for 10 min and stained with eBioscience Fixable Viability Dye eFluor780 (Thermo Fisher), according to manufacturer's procedure. Surface markers were stained for 30 min at 4°C at manufacturer's recommended dilution in 3% BSA/PBS. Cells were fixed and permeabilized using the eBioscience Foxp3 Transcription Factor staining buffer kit (Thermo Fisher) for 1 h according to manufacturer's procedure. Intracellular markers were stained for 30 min at 4°C at manufacturer's recommended dilution in 3% BSA/PBS. Antibodies used were: CD4-PE/Cy7 (clone RM4-5, Biolegend), CD8-BV480 (clone 53-6.7, BD Biosciences), NK1.1-SB600 (clone PK136, Thermo Fisher), FoxP3-eFluor450 (clone FJK-16s, Thermo Fisher), IFNγ-APC (clone XMG1.2, Biolegend), GATA3-BV711 (clone L50-823, BD Biosciences), RoRγt-BV650 (clone Q31-378, BD Biosciences), CD11c (clone BV480, BD Biosciences), CD11b (clone M1170, BD Biosciences), F4/80(clone BV711, Biolegend), and CD163 (clone PercCP/eF710, Thermo).

Cells were analyzed on an Attune NxT with autosampler (Thermo Fisher) by the ASU Knowledge Enterprise Core Research Flow Cytometry facility and data were processed with FlowJo v10.

### Statistical Analysis

Graphing and statistical analysis were performed using GraphPad Prism v8.4.3 (GraphPad Software, San Diego, CA, USA). Mean values were calculated for each analysis and are presented as mean ± SEM. Differences between groups were compared using analysis of variance (ANOVA), Fishers LSD (least significant difference) secondary analysis and unpaired Student's *T*-test. *P* < 0.05 were considered significant, represented in the figures as ^*^p < 0.05, ^**^p < 0.01, and ^***^p < 0.001.

## Results

### Native Serp-1 and PEGylated Serp-1 (Serp-1m5) Treatment Reduce Pristane-Induced DAH in C57/BL/6 SLE Mouse Model

**Figure 1:**
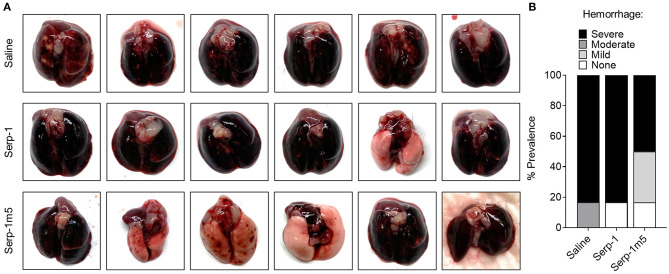
Serp-1 and its reengineered derivative Serp-1m5 reduce the prevalence of hemorrhage in pristane induced DAH mouse model. All mice induced by pristane were euthanized by CO_2_ on the 15th day of treatments with saline (6 mice), Serp-1 (6 mice), or Serp-1m5 (6 mice). Whole lungs from mice in each group were collected **(A)** and examined based on four grades of hemorrhage: severe, moderate, mild, or none **(B)**.

**Figure 2:**
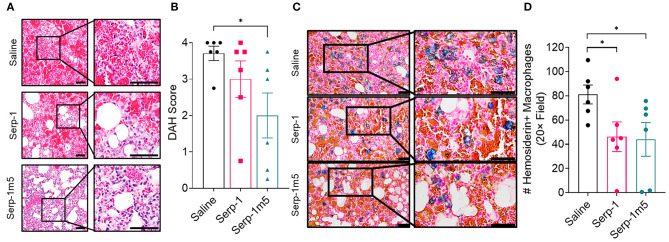
Histological analysis of DAH during treatment with saline control (*N* = 6 mice), GMP Serp-1 (*N* = 6 mice), or Serp-1m5 (*N* = 6 mice) proteins given daily. **(A)** Representative micrograph images of H&E stained lung sections at 14 days follow up. **(B)** DAH scores were evaluated by taking four 20× field of each section, and calculating the average score as standardized: 0, no hemorrhage; 1, 0–25% hemorrhage; 2, 25–50% hemorrhage; 3, 50–75% hemorrhage; 4, 75–100% hemorrhage. The DAH score of Serp-1m5 is significantly lower than that of the saline group (*p* = 0.0252). **(C)** Representative micrographs of Prussian blue staining at 14 days for each treatment group. **(D)** Both Serp-1 (*p* = 0.0370) and Serp-1m5 (*p* = 0.0424) significantly reduced detected hemosiderin laden macrophage counts in lungs form the pristane induced DAH model. *p < 0.05.

**Figure 3:**
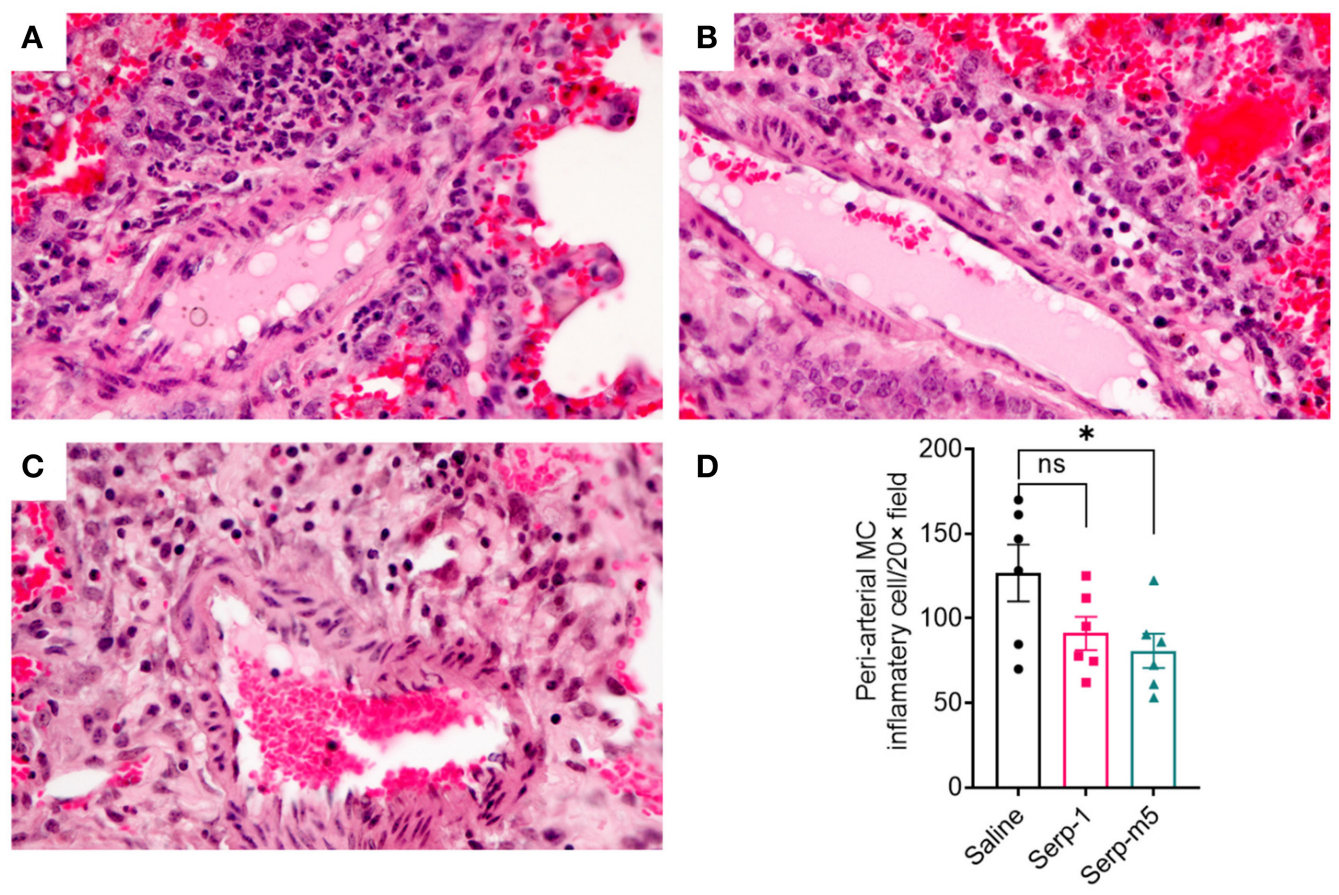
Saline treated mice had marked perivascular mononuclear cell infiltrates after pristane induction DAH **(A)**. Serp-1 treatment produced a non significant reduction in perivascular inflammatory cell counts **(B)**. Serp-1m5 treatment significantly reduced perivascular inflammatory cell counts when compared to the saline treated controls **(C)**. Perivascular mononuclear cell counts ± SEM are illustrated in panel **(D)**. *p < 0.05.

**Figure 4:**
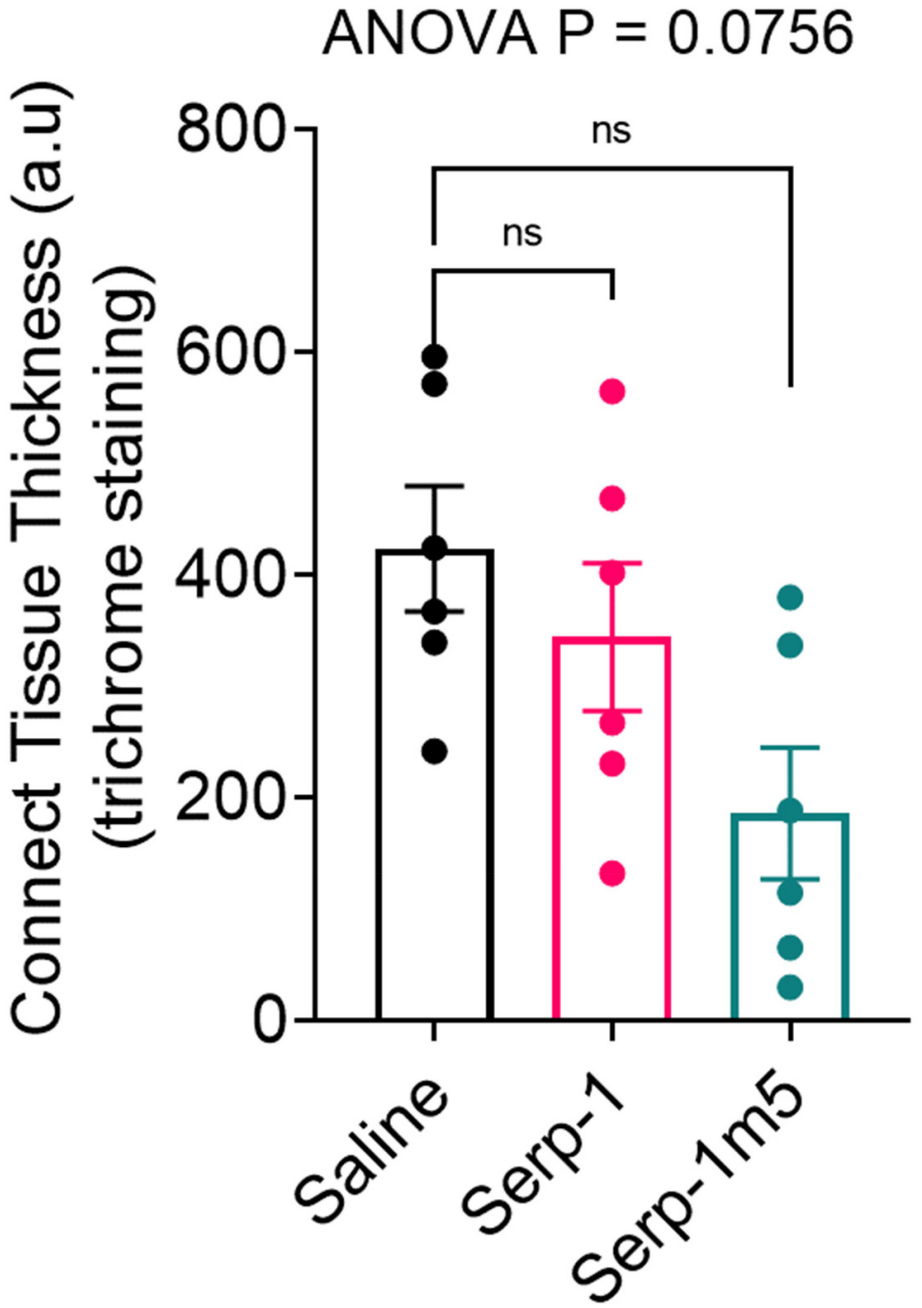
Trichrome staining indicated a nonsignificant increase in collagen staining in areas of hemorrhage in the saline treated controls. Serp-1 and Serp-1m5 treatment produced a non-significant decrease in fibrous tissue staining, here illustrated as measured thickness of fibrous tissue.

When each mouse was euthanized by CO_2_ inhalation, the lungs were immediately collected and imaged by a digital camera, before further processing (Figure 1A). All mice survived to 15 days post-pristane injections. According to the severity of the hemorrhage observed on gross pathology specimens, the lungs were divided into four grades: severe, moderate, mild, and no bleeding. As shown in Figure 1B, the whole lung pathology specimens demonstrate severe DAH in the saline treatment group, 5/6 severe DAH (5/6, *N* = 6) and 1 moderate DAH (1/6; *N* = 6). The Serp-1 treatment group contained five severe DAH (5/6; *N* = 6) and one non-DAH (1/6; *N* = 6). In the Serp-1m5 treatment group, three cases were severe (3/6; *N* = 6), two cases were mild (2/6; *N* = 6), and one case had no DAH (1/6; *N* = 6) (Figure 1; *p* = 0.2677). No hemorrhage was detected in normal healthy mice with or without Serp-1 treatment after euthanasia (Data not shown). Further evaluation of the hemorrhage was based on H&E staining and Prussian blue staining. Four 20 × fields were examined in randomly selected areas on the H&E sections for each mouse and scored according to the degree of bleeding as follows: 0, no hemorrhage; 1, 0–25% hemorrhage; 2, 25–50% hemorrhage; 3, 50–75% hemorrhage; 4, 75–100% hemorrhage. Representative histology images are presented in Figure 2A. The average DAH score for each mouse was calculated. Measurements were performed by two independent experimenters blinded to the treatments given to pristane injected mice. On histological examination, the DAH score of the Serp-1 treatment group indicates a trend toward a reduction and the DAH score for the Serp-1m5 treatment group is significantly lower than that of the saline group (Figure 2B; Serp-1, *p* = 0.2172; Serp-1m5, *p* = 0.0252). Prussian blue staining was used to detect hemosiderin laden macrophages. Serp-1 and Serp-1m5 treatments both significantly reduced detected hemosiderin laden macrophage cells when they were compared to the saline treatment group (Figures 2C,D; Serp-1, *p* = 0.0370; Serp-1m5, *p* = 0.0424). On H&E stained sections, there was a significant reduction in perivascular mononuclear cell infiltrates with Serp-1m5 treatments and a trend for Serp-1 treatment (Figure 3) (ANOVA, *P* = 0.0104; Serp-1m5, *p* = 0.0026, Serp-1, *p* = 0.1215). Trichrome staining revealed a trend toward a reduction in collagen or fibrous tissue staining around areas of excess hemorrhage, with both Serp-1m5 and Serp-1 treatments (Figure 4).

### Serp-1 and Serp-1m5 Treatment Reduced M1 Macrophages and Neutrophils in Pristane-Induced DAH Model

**Figure 5:**
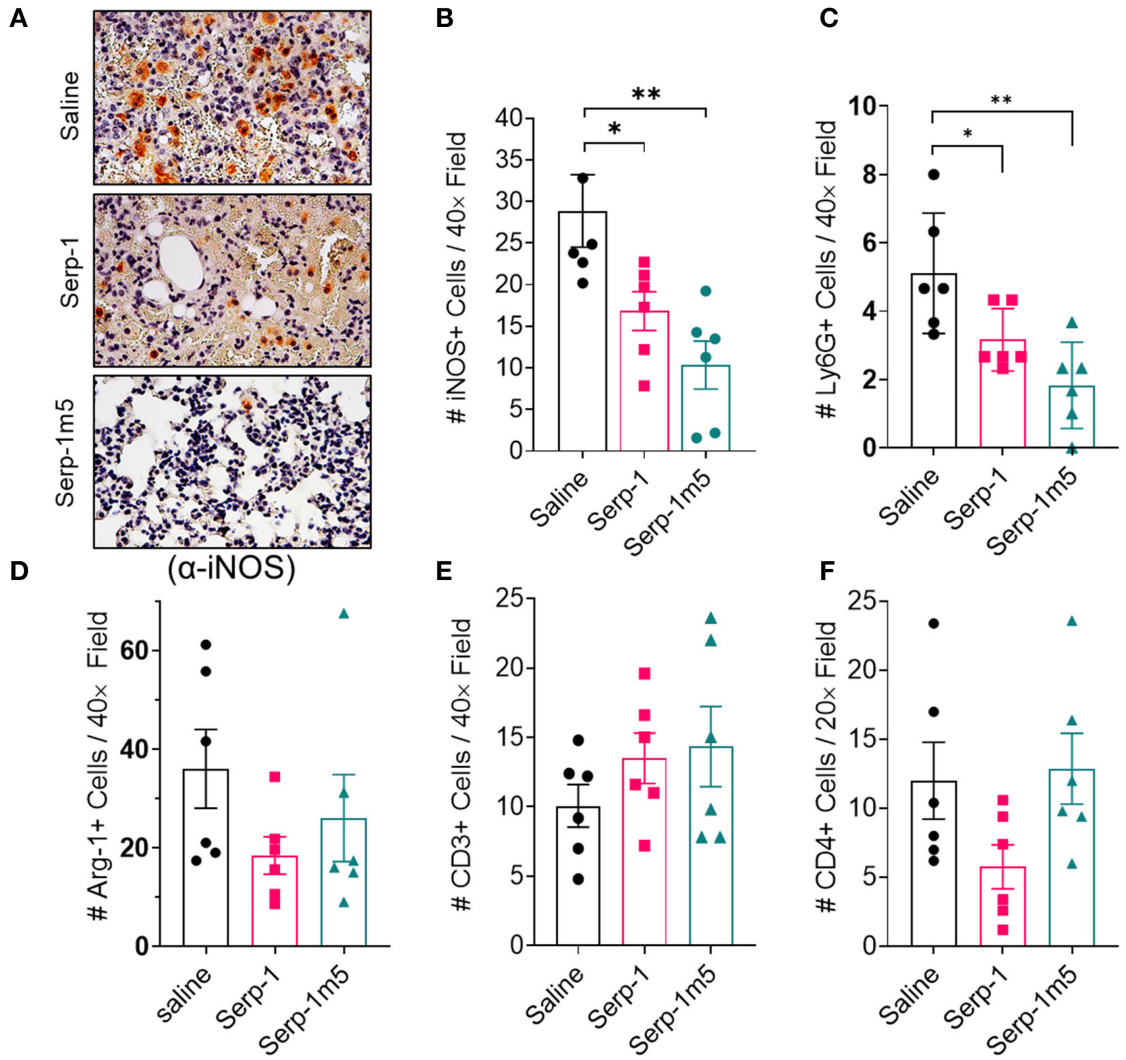
DAH Serp-1 and Serp-1m5 treatments reduced M1 macrophage and neutrophil numbers in lung sections after pristane-induced DAH, but did not significantly affect M2 macrophage counts, CD3+ cell, or CD4+ cell infiltration. **(A)** Representative micrographs (40×) of iNOS + IHC staining in lungs at 14 days post-pristine injection. **(B)** Serp-1 and Serp-1m5 group had significantly lower numbers of M1 macrophage counts marked by positive staining for iNOS IHC staining (Serp-1, *p* = 0.0350; Serp-1m5, *p* = 0.0053) when compared to the saline treatment group. **(C)** The Serp-1m5 treated group had significantly lower numbers of Ly6G+ neutrophils (Serp-1, *p* = 0.0371; Serp-1m5, *p* = 0.004). Serp-1 treatment had a strong trend toward reduced neutrophil counts, but did not reach significance. The number of Arg-1 + M2 macrophages **(D)**, CD3+ T cells **(E)**, or CD4+ T cells **(F)** were not statistically different among the three groups, although a non-significant trend toward increased Arg+ cells was seen. *p < 0.05, **p < 0.01.

The DAH in the pristane induced mouse model is reported to be macrophage dependent (13). We characterized the proinflammatory M1 macrophage polarization in the mouse lung tissue sections by IHC staining for iNOS (iNOS+). As shown in Figure 5, the lungs of mice treated by Serp-1 and Serp-1m5 treatments had significantly lower numbers of iNOS+ M1 macrophages than that of saline control (Figures 5A,B; Serp-1, *p* = 0.0350; Serp-1m5, *p* = 0.0053). Additionally, lung tissues treated with Serp-1 and Serp-1m5 also have significantly less detected numbers of Ly6G+ neutrophils than the saline treatment group (Figure 5C; Serp-1, *p* = 0.0371; Serp-1m5, *p* = 0.004). We also performed IHC staining for arginase-1 (Arg-1) to characterize the anti-inflammatory M2 macrophages (Figure 5D), but no statistical differences among these three groups were observed for Arg-1+ M2 macrophage. Arg-1+ M2 macrophage staining detected a nonsignificant trend toward increased numbers. Serp-1 and Serp-1m5 treatment groups have a trend to increased CD3+ T cells (Figure 5E) when compared to saline treated mice but this does not achieve significance (*p* = 0.3595). There were no identified changes in different in CD4+ T helper cell staining either (Figure 5F; *p* = 0.1015).

### Serp-1m5 Treatment Reduced Macrophage Counts on Flow Cytometry Analysis in Cell Isolates From the Spleen of Mice After 15 Days of Induction With Pristane

**Figure 6:**
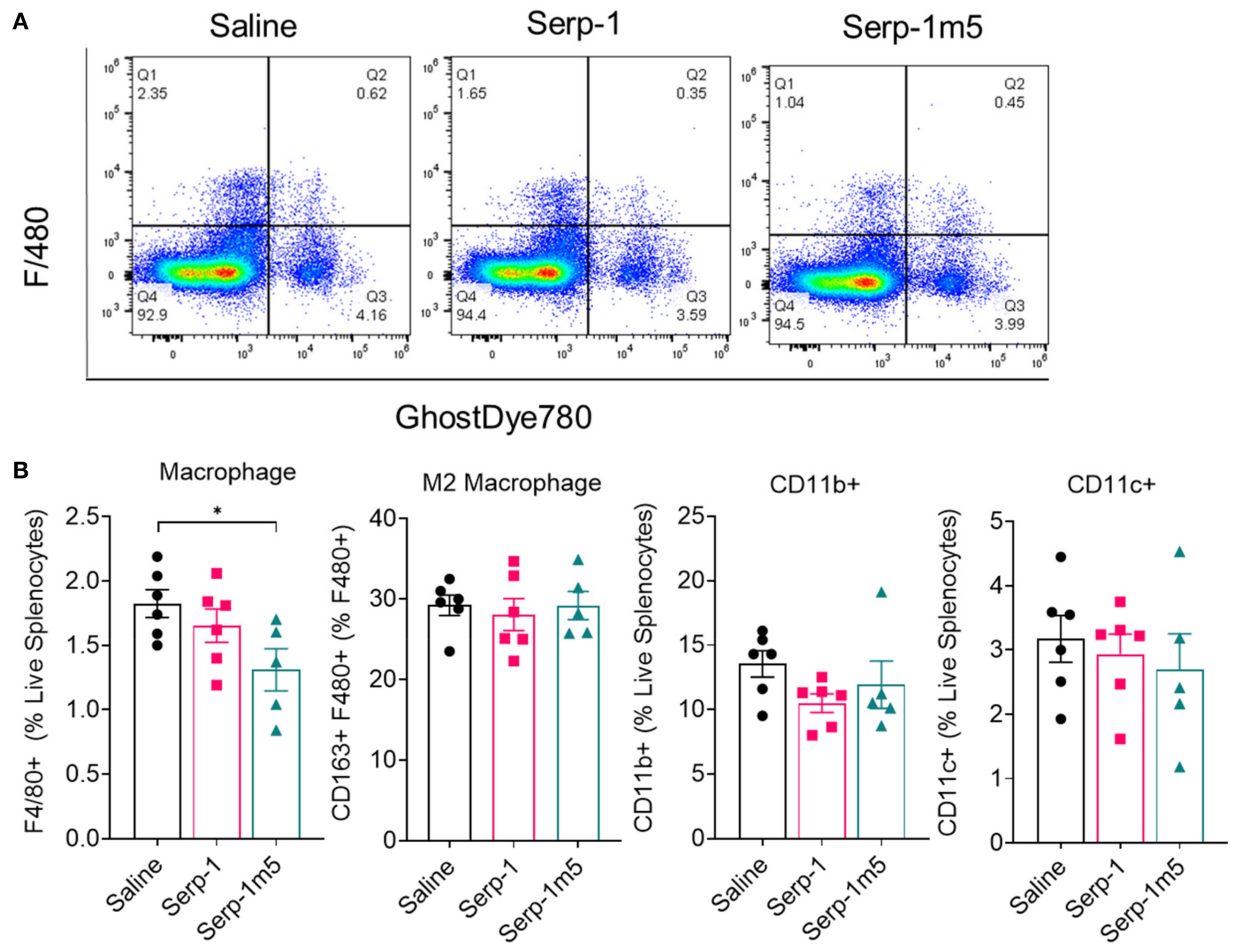
Serp-1m5 treatment reduces macrophages in the spleen of mice after 15 days of induction with pristane. **(A)** Representative images of flow cytometry for F4/80+ macrophages in live splenocytes demonstrating reduced F4/80+ cells with Serp-1m5 treatment. **(B)** Flow cytometry of macrophages demosntrated decreased frequency of F4/80+ macrophages (*p* = 0.0173) in live splenocytes in Serp-1m5 treated mice compared with saline treated mice while no significant differences in the frequency of CD11b+cells, CD11c+ cells in living spleen cells and CD163+F480+M2 macrophages in F480+ macrophages among the three groups. *p < 0.05.

**Figure 7:**
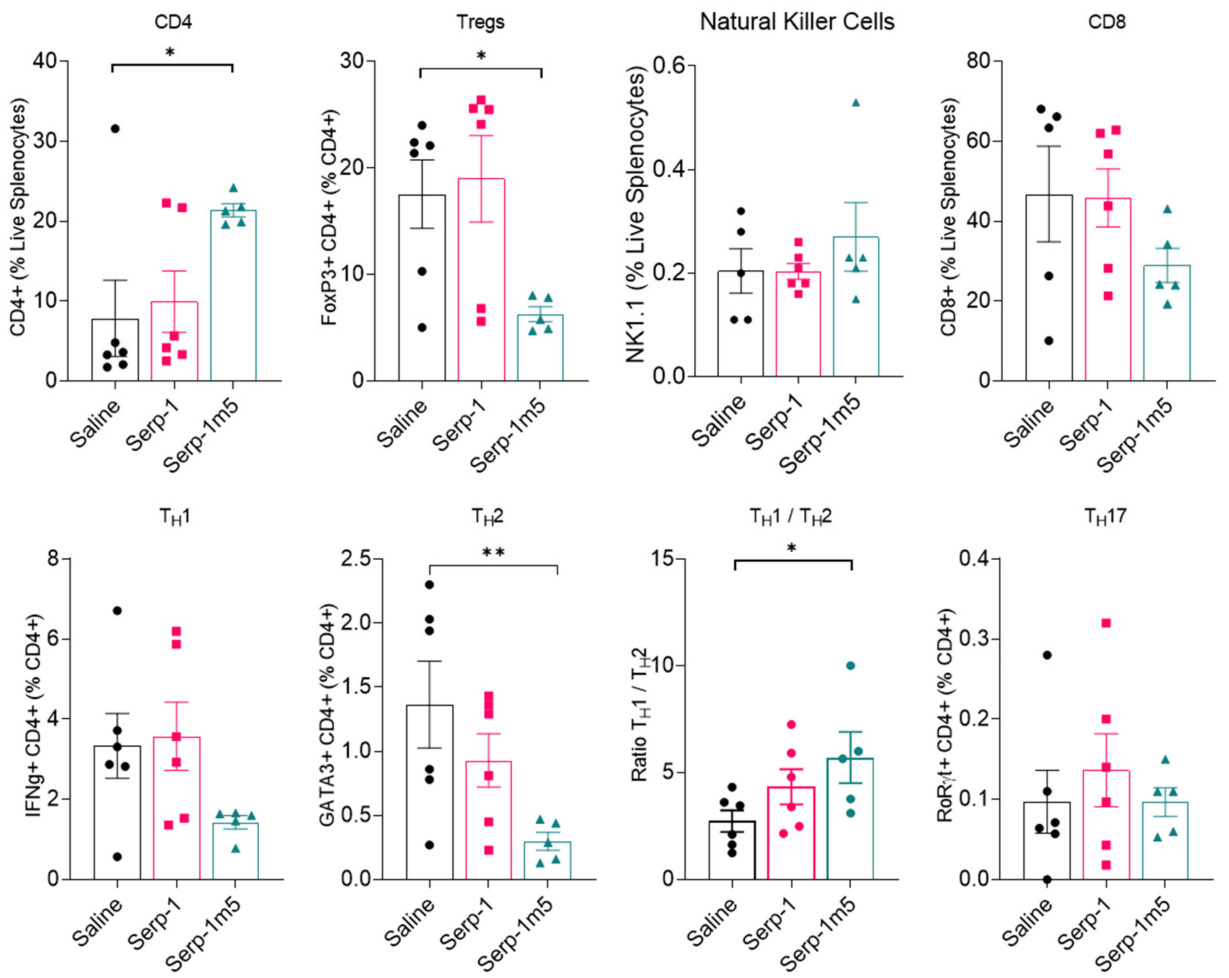
Flow cytometry of lymphocytes indicate a decreased frequency of GATA3+CD4+ Th2 cells (*p* = 0.0097), Tregs cells (*p* = 0.0285) in CD4+ T cells and increased frequency of CD4+ T cell in live splenocytes (*p* = 0.0268), ratio of Th1/Th2 (*p* = 0.0287) in Serp-1m5 treated mice compared with saline treated mice while no significant differences in the frequency of NK cells, CD8+ T cells, IFNg+CD4+ Th1 cells, Th17 cells in CD4+ T cells among the three groups. *p < 0.05, **p < 0.01.

In order to examine the potential systemic immune cell responses to Serp-1 and Serp-1m5 treatments on mice after pristane induction of DAH, we examined splenocyte isolates from each mouse at 14 days follow up. We did not observe consistent changes in spleen size among these three groups when we collected mouse tissues after 15 days of induction with pristane (17 mice in total–6 with saline, 6 with Serp-1, and 5 with Serp-1m5 treatment; the spleen from one mouse in the Serp-1m5 group was not collected and was not tested by flow cytometry). Flow cytometry analysis of splenocytes demonstrated significantly decreased F4/80+ macrophages (Figure 6; *p* = 0.0173) in live splenocytes from Serp-1m5 treated mice. No change in detected CD163+ M2 in F4/80+ macrophages, nor in CD11b or CD11c cells in live splenocytes was seen (Figure 6B). A significant increase in CD4+ T cells in live splenocytes (*p* = 0.0268) and the TH1/Th2 ratio (*p* = 0.0287) was detected (Figure 7); A significant decrease in Tregs (*p* = 0.0285) and GATA3+CD4+ Th2 cells (*p* = 0.0097) in CD4+ T cells were detected with Serp-1m5 but not the unmodified Serp-1 (Figure 7); There was no significant change in the frequency of NK cells, CD8+ T cells, CD11b+cells, CD11c+ cells in living spleen cells, IFNg+CD4+ Th1 cells, Th17 cells in CD4+ T cells, and CD163+F480+M2 macrophages in F4/80+ macrophages among the three groups (Figures 6, 7).

### Serp-1 and Serp-m5 Reduce C5b/9 Complement Positive Cell Counts in DAH Model Lung Sections

**Figure 8:**
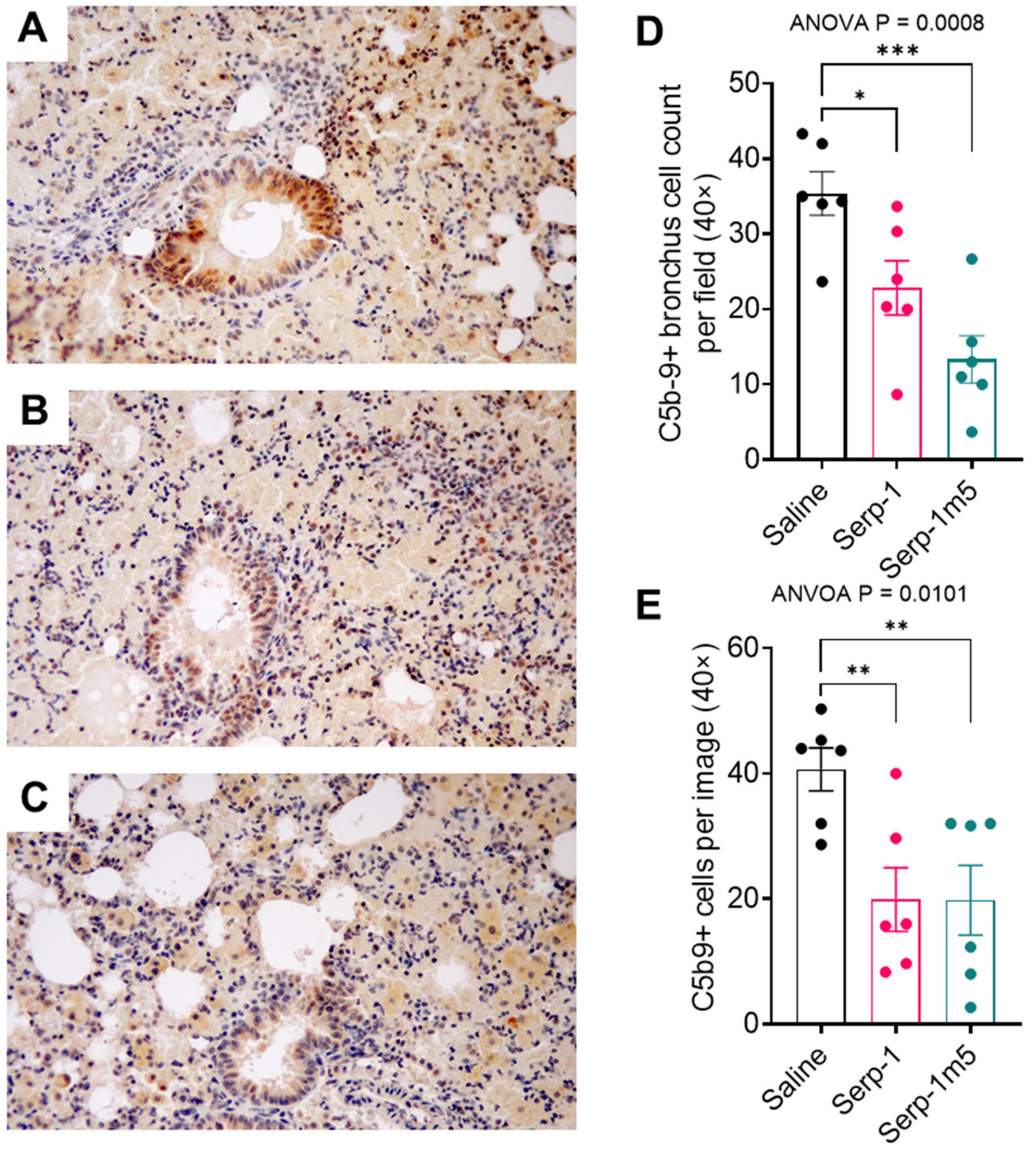
C5b/9 staining is markedly increased in the bronchial epithelium and in the surrounding lung parenchyma in saline treated mice with pristane induced DAH **(A)**. Serp-1 **(B)** and Serp-1m5 **(C)** significantly detected reduced bronchial staining. Bar graphs demonstrate significant changes in the mean cell counts ± SEM for cells positively stained for C5b/9 in the bronchial epithelium **(D)** and in the lung parenchyma **(E)**; Bronchus cell counts—*p* = 0.0008 ANOVA; Parenchyma ANOVA—*p* = 0.0101. *p < 0.05, **p < 0.01, ***p < 0.001.

Serp-1 pulled down human plasma C3 and vitronectin as determined by mass spectrometry. C5b/9 is a final stage in complement activation, the membrane attack complex. We have demonstrated a significant reduction in cells staining positively in both bronchial (ANOVA *p* = 0.0008) and parenchymal tissues (ANOVA *p* = 0.0101) in lung sections from mice treated. Both Serp-1 and Serp-1m5 produced significant reduction; Serp-1, bronchus *p* = 0.0146; parenchyma *p* = 0.0077 and Serp-1m5, brochus *p* = 0.0002, parenchyma *p* = 0.0074; Figure 8).

### Serp-1 and Serp-1m5 Treatment Reduced Cell Membrane Free Soluble uPAR in the Lung Tissue of Pristane-Induced DAH Mouse Model

**Figure 9:**
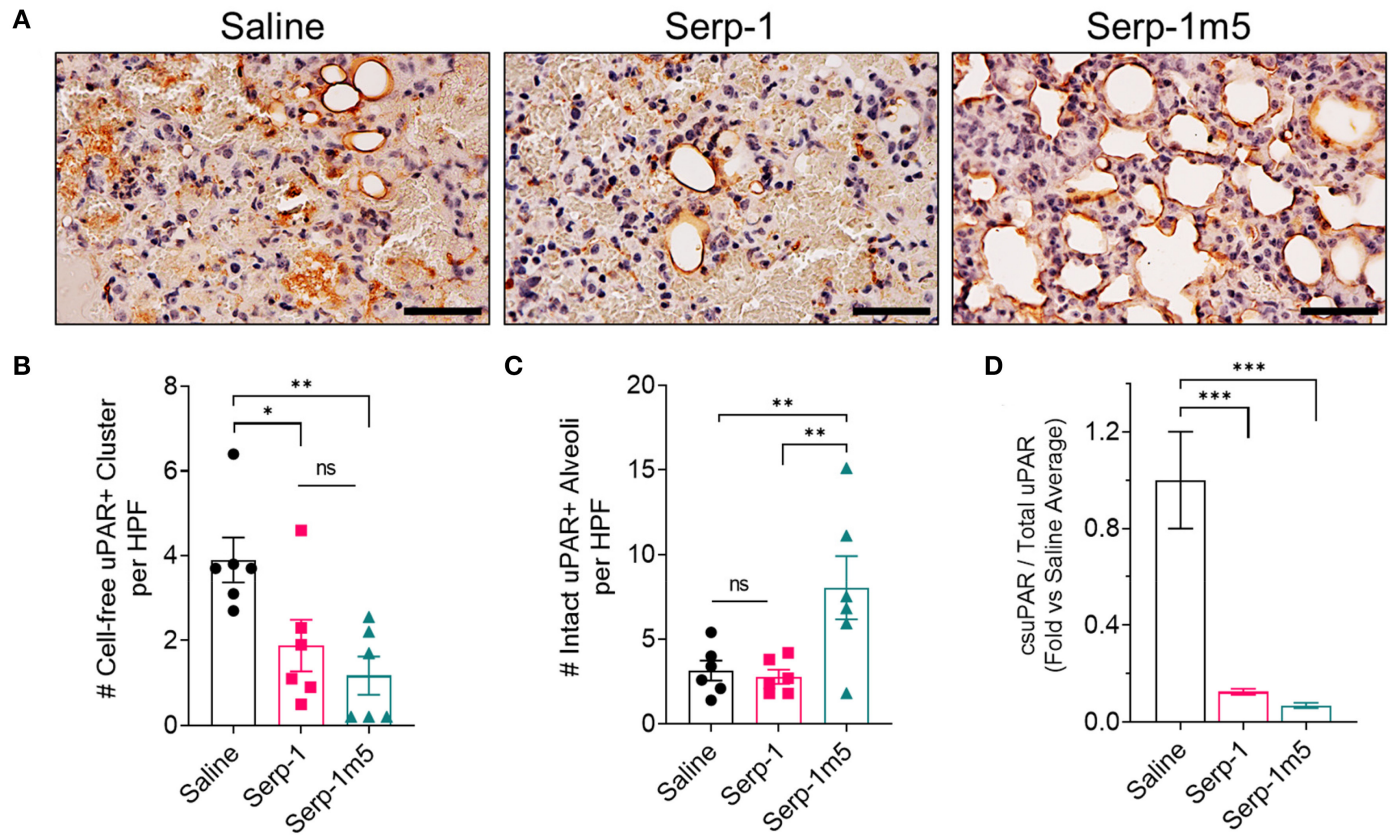
Serp-1 and Serp-1m5 treatment reduce soluble uPAR in the pristane-induced DAH model. **(A)** The representative pictures of uPAR IHC staining. **(B)** Serp-1 and Serp-1m5 reduced cell-free uPAR+ clusters in IHC staining when compared to the saline treatment group (Serp-1, *p* = 0.0172; Serp-1m5, *p* = 0.0025). **(C)** Serp-1m5 increased intact uPAR+ stained alveoli along the inner rim when compared to the saline treatment group (*p* = 0.0091; Serp-1m5 vs. saline). **(D)** The ratio of csuPAR to total uPAR of Serp-1 and Serp-1m5 tested by ELISA of lung tissue was significantly lower than that of the saline group (Serp-1, *p* = 0.0004; Serp-1m5, 0.0002). *p < 0.05, **p < 0.01, and ***p < 0.001.

The uPA-uPAR interaction plays an important role during inflammatory cell invasion and activation. It has been reported earlier this year that cell free uPAR, i.e., soluble uPAR (suPAR), is related to organ damage in SLE patients (31). Our research has previously demonstrated that the immune modulation produced by Serp-1 in aortic allografts as well as Serp-1 inhibition of macrophage activation and diapedesis in tissue culture is dependent on the uPAR (25). In prior work, Serp-1 lost its ability to reduce inflammation and to reduce plaque growth in uPAR knock out aortic allograft transplants in mouse models (18). The depletion of uPAR also abolished the function of Serp-1 to promote wound healing (30). We therefor performed IHC staining for uPAR to characterize uPAR expression after pristine induction, with or without serpin treatments. It can be seen from the IHC staining of lung tissue that Serp-1 and Serp-1m5 reduce non-specific uPAR+ clusters (Figures 9A–C; Serp-1, *p* = 0.0172; Serp-1m5, *p* = 0.0025), detected as non-cell associated clumps of positive staining in the lungs, when compared to the saline treatment group. In contrast, there was increased detection of intact uPAR+ stained alveoli along the inner rim (Figures 9A–C; Serp-1m5 vs. saline, *p* = 0.0091). Control experiments without primary antibody did not detect nonspecific staining. To further quantitatively analyze the dissociation of uPAR, we compared the uPAR extracted from tissue without detergent (PBS only) to the total uPAR extracted with RIPA buffer containing 0.1% SDS. ELISA was performed to determine the concentration of uPAR in each extraction. We set the average ratio of lung tissue cell membrane free uPAR (csuPAR) to total uPAR in each lung treated with saline as one and normalized all the ratios of csuPAR/total uPAR (Figure 9D). The ratio of csuPAR to total uPAR of Serp-1 and Serp-1m5 groups was significantly lower than that of the saline treated group (Serp-1, *p* = 0.0004; Serp-1m5, *p* = 0.0002). This data confirmed our observation in the IHC staining to uPAR (Figures 9A–C).

## Discussion

The pathogenesis of DAH is reported to be caused by defects in macrophage phagocytic function and reduction in the removal of apoptotic cells. Apoptotic fragments activate autoreactive B cells and T cells, leading to the production of autoantibodies and the formation of circulating immune complexes (ICs). ICs are then believed to activate the classical complement pathway, thereby causing pulmonary capillary vasculitis, damage to the basement membrane, and capillary leakage with extravasation of red blood cells (RBC) and bleeding into the alveolar cavity. This continuous auto-Ab-mediated enhancement of the complement system also causes complement depletion and reduces the ability of phagocytes to remove dead cell debris, thus initiating a vicious circle (32). Intraperitoneal injection of pristane in B6 mice is an accepted model for lupus that simulates the SLE DAH pathological process (14, 33, 34). After injection, pristane migrates to the lungs, resulting in increased cell death, activation of inflammatory cells such as macrophages, activation of the classical pathway of complement and ICs formation and additionally alveolar hemorrhage similar to human DAH occurs. Studies have demonstrated that this process is macrophage dependent in the mouse DAH model. Neutrophil depletion is however reported to not be protective in prior mouse model studies when using antibody to neutrophil elastase. In contrast treatment with clodronate liposomes (CloLip) to reduce macrophages was able to prevent DAH (13). Cell debris depends on the opsonization of natural IgM, C3, and CR3/CR4 on the surface of macrophage cells to activate downstream inflammatory pathways. CD11b^−/−^ mice are protected against the development of pristane-induced DAH (5), and mice with C3 deficiency and CD18 deficiency are also resistant. Reduction of complement in wild-type mice by cobra venom factor (CVF) can prevent DAH (13), while antibody to suppress neutrophils was not effective.

From prior research, it is understood that macrophages play a decisive role in the SLE DAH pathological process. Our research has demonstrated that the administration of Serp-1 or Serp-1m5 after intraperitoneal injection of pristane can significantly reduce the occurrence and severity of DAH (Figures 1, 2). We performed immunohistochemistry using typical markers of M1(iNOS), M2 (ARG1) macrophages and neutrophils (Ly6G). Quantitation of stained cells demonstrated that Serp-1 and Serp-1m5 treatments significantly reduced M1 macrophage polarization and neutrophil infiltration in the lungs of the DAH mouse model. Flow cytometry analysis of spleen cells was also consistent with the findings on immunostaining in the lungs. Flow cytometry analysis demonstrated that the macrophage isolates from the spleen of Serp-1m5 treated mice after pristane induction were significantly reduced when compared to saline treated pristane injected control mice. In contrast, the number of M2 macrophages did not change significantly. Therefore, it is deduced that the efficacy of Serp-1 and Serp1m5 treatment is associated with a significant reduction in M1 macrophage. Variable changes in lymphocytes counts on IHC with some significant differences in CD4, Treg, and Th2 counts on flow cytometry were observed and on flow cytometry. These T cell changes were limited to Serp-1m5 treatments. These findings are consistent with previous studies with Serp-1 suggesting a more significant effect on macrophage responses. However, the more pronounced effects of Serp-1m5 both on lung hemorrhage, M1 macrophage and Ly6G counts on IHC and also greater effects of Serp-1m5 on splenocyte analyses might suggested that some of the enhanced activity of the Serp-1m5 protein is due to a larger effect overall on immune cell responses in the DAH model in mice.

M1 macrophages are involved in the pathogenesis of various autoimmune inflammatory diseases, including multiple sclerosis, rheumatoid arthritis, inflammatory bowel diseases, asthma, and SLE (1, 35–39), we have proposed that targeting M1 cells with Serp-1 treatment will provide a potential treatment for autoimmune diseases, suggesting that Serp-1 can inhibit the activation of macrophage M1 to protect SLE patients against the development of DAH. Serp-1 is a proven inhibitor of activated serine proteases, functioning to bind, and inhibit both tPA and uPA as well as thrombin and fXa as noted in the introduction. The thrombotic and thrombolytic cascades also activate immune and inflammatory cell responses and conversely platelets and thrombosis are activated on the surfaces of dysfunctional endothelial cells and/or activated macrophages in the arterial wall. uPA and tPA are plasminogen activators, serine proteases, in the thrombolytic, clot dissolving cascade, and can initiate matrix metalloproteinase activation and connective tissue degradation allowing immune cell invasion. Complement is also serine protease central to immune cell responses. We have posited that Serp-1 inhibits the migration of M1 macrophages to the lung via blockade of the uPA/uPAR complex on the surface of activated inflammatory phagocytes. Activated macrophage expression of uPA and tPA leads to the activation of MMPs allowing cell invasion into damaged tissues by breaking down surrounding connective tissue or the arterial endothelial glycocalyx. It is, however, not known whether Serp-1 may directly promote macrophage polarization to a M2 phenotype. The uPAR is part of a large lipid raft that interacts with many cell surface integrins and low density lipoprotein related protein receptor (LRPR) as well as chemokine receptors. We had previously demonstrated that Serp-1 alters macrophage migration *in vitro* via uPAR and filamin B (an actin binding protein) dependent mechanisms. Our previous studies have demonstrated that Serp-1 can reduce monocyte/ macrophage adhesion and migration across endothelial monolayers *in vitro* and into mouse ascites *in vivo*. Serp-1 applied to monocytes alters the expression of filamin B and CD18, increasing filamin B and decreasing CD-18 expression. These alterations in gene expression are uPAR dependent and application of siRNA to filamin blocked Serp-1 mediated inhibition of monocyte migration *in vitro*. Filamin b and uPAR are co-localized and co-immunoprecipitated with Serp-1 (25). Therefore, we would suggest that Serp-1 exerts its anti-inflammatory effects by modifying uPAR-CD18 and filamin b in monocytes to mediate the decrease of M1 macrophages in lung tissue. We would like to further note that in prior reported studies of the mechanisms of DAH development, DAH was resistant to CD18-deficient mice (5).

Serp-1 treatment may also have the added benefit of reducing activation of fibrinolysis and thus may directly mediate a reduction in bleeding. The uPA/uPAR complex is traditionally considered to act predominantly on cellular activation and immune cell responses rather than as a primary regulator of thrombolysis. tPA, the fibrinolytic serine protease that Serp-1 also inhibits, is considered the central mediator of the regulation of clot lysis in the blood stream. In this study, our immunohistochemical study of uPAR in lung tissues demonstrated that when compared with saline treated DAH mice, the soluble uPAR in the Serp-1 treatment group was significantly reduced, while uPAR on the intact alveolar cells was preserved significantly. This is consistent with the uPAR ELISA analysis of lung tissue that similarly detected Serp-1 reductions in soluble uPAR. uPAR is composed of three homologous domains and is connected to the cell surface through glycosylphosphatidylinositol (GPI) anchors. The three domains of uPAR include the main uPA (40), and the extracellular matrix protein vitronectin binding site (41). uPAR is easily cleaved by several proteases, including physiologically related enzymes such as neutrophil elastase, plasmin and uPA itself (42–44).

Soluble uPAR is currently considered to be a biomarker of inflammation and immune system activation but has not to date been examined nor associated with DAH in SLE patients. Elevated suPAR levels are associated with a variety of inflammatory diseases, such as systemic inflammatory response syndrome (SIRS), cancer, local segmental glomerulosclerosis, cardiovascular disease, type 2 diabetes, asthma, liver failure, COVID, and SLE (31, 45–50). After DAH occurs, the alveolar basement membrane tissue is destroyed by immune complex (IC) deposition and inflammatory cell adhesion, red blood cells then extravasate (leak out of damaged vessels), and abnormal blood coagulation pathways are initiated. Cleavage of uPAR by plasmin and uPA causes the release of soluble uPAR from the cell surface membrane into surrounding tissues, weakening of the anchoring of cells to the extracellular matrix, reducing endothelial cell connections and, at the same time, triggers a series of proteolytic cascade reactions to degrade the components of the extracellular matrix leading to further destruction of alveolar tissue structure and increased bleeding. Therefore, Serp-1 can reduce the cleavage of uPAR, maintain cell adhesion to the extracellular matrix as well as reducing inflammatory cell invasion via inhibiting uPA and plasmin and activation of matrix metalloproteinases. As we have seen in our experiments, soluble uPAR is significantly reduced, and uPAR in intact alveolar tissue cells increased. Meanwhile, Serp-1 reduces the downstream protease cascade by modulating the coagulation and fibrinolysis process, thereby preventing further destruction of lung tissue structure and pulmonary hemorrhage. Activation of complement is also central to immune responses. We have demonstrated Serp-1 binding to C3 in human plasma by MS analysis. We also report here a reduction in C5b/9 IHC complement staining with serpin treatments, which may be secondary to efficacy in reducing uPA/uPAR activity or conversely complement. Prior work would suggest that uPAR is a principal target. However, binding and modulation of a second pathway via C3 may also contribute to the benefits seen with serpin treatments in this lupus DAH model. These findings support a reduction in overall immune cell activation with serpin treatment but do not provide a final proof of mechanism. The studies do suggest a close correlation between uPAR expression, as well as complement activation, both serine protease pathways, and serpin efficacy in this mouse model of pristane induced SLE DAH.

In summary, Serp-1 and PEGylated Serp-1 (Serp-1m5) effectively reduce the frequency and severity of pristane-induced diffuse pulmonary hemorrhage in mice through multiple factors, while the modified Serp-1 protein has greater efficacy than its wild type does in this experimental DAH model. This research proves that Serp-1 and its derivates are very promising candidates of therapeutics for DAH, which has no proven effective treatment available now. In this direction, the molecular mechanism under the therapeutic effectiveness to DAH and the technologies for the modification will be further studied.

## Data Availability Statement

The original contributions presented in the study are included in the article/supplementary material, further inquiries can be directed to the corresponding author/s.

## Ethics Statement

The animal study was reviewed and approved by Biodesign Institute, ASU Institutional Animal Care and Use Committee Animal Protocol Review ASU Protocol Number: 20-1761R RFC 1.

## Author Contributions

QG, JRY, LZ, and ARL designed the study, LZ and QG developed materials. LZ, QG, and JRY performed the animal studies and histology assays, RB and TO assisted in the protein purification, KB, EA, MB, and LNS performed histology, JW and PF provided scientific feedback and discussion, QG, LZ, JRY, and SL analyzed the data, QG, ARL, and LZ wrote the manuscript. All authors reviewed the manuscript.

## Conflict of Interest

The method of Serp-1 modification has been submitted for a provisional patent application with title of “New composition of immunomodulating Serpin, Serp-1” (PCT Application No. 63/017,598). ARL, LZ, QG, JRY, and JW are inventors of this patent. JW is employed by Exalt Therapeutics but has received no funding for his work on this project nor the research presented in this manuscript. The remaining authors declare that the research was conducted in the absence of any commercial or financial relationships that could be construed as a potential conflict of interest.
